# Ectopic Expression of Executor Gene *Xa23* Enhances Resistance to Both Bacterial and Fungal Diseases in Rice

**DOI:** 10.3390/ijms23126545

**Published:** 2022-06-11

**Authors:** Zhiyuan Ji, Hongda Sun, Yena Wei, Man Li, Hongjie Wang, Jiangmin Xu, Cailin Lei, Chunlian Wang, Kaijun Zhao

**Affiliations:** National Key Facility for Crop Gene Resources and Genetic Improvement (NFCRI), Institute of Crop Sciences, Chinese Academy of Agriculture Sciences (CAAS), Beijing 100081, China; jizhiyuan@caas.cn (Z.J.); hongda_sun1995@163.com (H.S.); wingqianye@163.com (Y.W.); 13230888788@163.com (M.L.); 15703411106@163.com (H.W.); xjm199208@163.com (J.X.); leicailin@caas.cn (C.L.)

**Keywords:** rice, executor gene, *Xanthomonas oryzae*, *Magnaporthe oryzae*, transcription activator-like effectors

## Abstract

Bacterial blight (BB) and bacterial leaf streak (BLS), caused by phytopathogenic bacteria *Xanthomonas oryzae* pv. *oryzae* (*Xoo*) and *Xanthomonas oryzae* pv. *oryzicola* (*Xoc*), respectively, are the most serious bacterial diseases of rice, while blast, caused by *Magnaporthe oryzae* (*M*. *oryzae*), is the most devastating fungal disease in rice. Generating broad-spectrum resistance to these diseases is one of the key approaches for the sustainable production of rice. Executor (*E*) genes are a unique type of plant resistance (*R*) genes, which can specifically trap transcription activator-like effectors (TALEs) of pathogens and trigger an intense defense reaction characterized by a hypersensitive response in the host. This strong resistance is a result of programed cell death induced by the *E* gene expression that is only activated upon the binding of a TALE to the effector-binding element (EBE) located in the *E* gene promoter during the pathogen infection. Our previous studies revealed that the *E* gene *Xa23* has the broadest and highest resistance to BB. To investigate whether the *Xa23*-mediated resistance is efficient against *Xanthomonas oryzae* pv. *oryzicola* (*Xoc*), the causal agent of BLS, we generated a new version of *Xa23*, designated as *Xa23p1.0*, to specifically trap the conserved TALEs from multiple *Xoc* strains. The results showed that the *Xa23p1.0* confers broad resistance against both BB and BLS in rice. Moreover, our further experiment on the *Xa23p1.0* transgenic plants firstly demonstrated that the *E*-gene-mediated defensive reaction is also effective against *M*. *oryzae*, the causal agent of the most devastating fungal disease in rice. Our current work provides a new strategy to exploit the full potential of the *E*-gene-mediated disease resistance in rice.

## 1. Introduction

Rice is a major staple food crop that feeds half of the world’s population. Bacterial blight (BB) and bacterial leaf streak (BLS), caused by *Xanthomonas oryzae* pv. *oryzae* (*Xoo*) and *Xanthomonas oryzae* pv. *oryzicola* (*Xoc*), respectively, are the most serious bacterial diseases of rice [1,2]. The two diseases, especially BB, cause severe yield losses that increase food insecurity in the world. Both *Xoo* and *Xoc* use transcription activator-like effectors (TALEs) as major virulence factors to promote rice disease susceptibility [3,4]. During the infection, TALEs are translocated into host plant cells by the bacterial type III secretion system and function as eukaryotic transcription factors in the nucleus, where they drive the expression of host susceptibility (*S*) genes via binding to the effector-binding element (EBE) in the promoters [5,6].

To counter the attack from TALEs, plants have evolved the so-called executor (*E*) genes, a unique type of resistance (*R*) genes identified in the plant–*Xanthomonas* interaction systems [7,8]. The *E* genes can specifically trap TALEs by using the evolved EBEs and triggering an intense plant defense reaction characterized by a hypersensitive response (HR) [9,10,11,12]. To date, four *E* genes, *Xa7*, *Xa10*, *Xa2,3* and *Xa27*, have been cloned from rice, and they have displayed different resistance spectrums to BB due to the different TALEs trapped [8,13]. However, no natural *E* gene for BLS has been discovered, even though *Xoc* harbors the largest number of TALEs in phytopathogenic bacteria [14]. It has been explained that *Xoc* could suppress host resistance in multiple ways [15,16,17].

Despite the close relationship between *Xoo* and *Xoc*, few *R* genes can confer resistance to both diseases BB and BLS [1,18,19]. The *Xa1* allelic genes, which are nucleotide-binding leucine-rich repeat (NLR)-type *R* genes in rice, initiate a strong resistance response by recognizing typical TALEs in *Xoo* and *Xoc* [20,21,22]. Unfortunately, the broad resistance mediated by *Xa1* allelic genes was defeated by interfering TALEs (iTALEs); even some *Xa1* allelic genes, such as *Xa14*, can avoid the suppression of type B iTALEs [20]. To successfully induce host susceptibility (*S*) genes for disease development, TALEs need to hijack the basal transcription factor (TF) TFIIAγ5 (XA5) through the transcription factor binding (TFB) region [23,24]. The mutation type of TFIIAγ5, xa5 encoded by the recessive disease resistance allele of *Xa5*, arrests TALE-induced susceptibility by attenuating the binding affinities of TALEs to a host TF. However, TALEs can recruit another TF, namely, OsTFIIAγ1, to partially compensate for the attenuated susceptibility. *xa5* provides race-specific or partial resistance to *Xoo* and *Xoc* [25,26,27].

**Figure 1:**
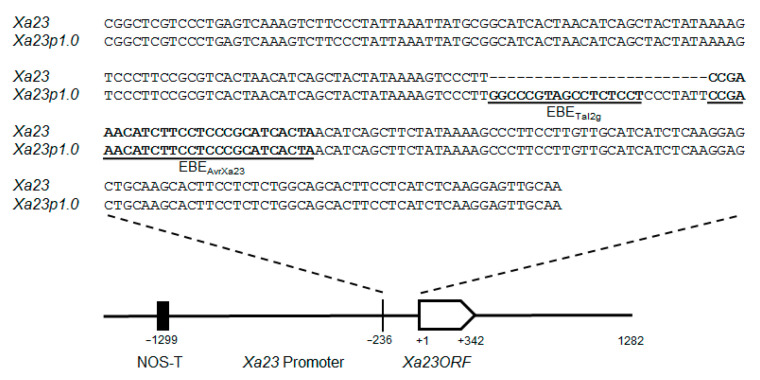
Schematic map and nucleotide sequences of generated *Xa23p1.0*. A 2.5 kb fragment containing the *Xa23* locus was modified by inserting the 17 bp EBE_Tal2g_ and linker sequence CCCTAT into the promoter region via Gibson cloning.

The TALE–EBE interaction mechanisms have been decoded by two research groups. The repeat variable di-residues (RVDs) in every repeat in the central TALE region can specifically recognize one base of target DNA [28,29]. Based on the RVD-EBE recognition codes, artificial EBEs can be integrated into *E* genes to engineer broad and durable *R* genes for multiple *Xanthomonas* diseases [30,31,32]. A single modified *E* gene could realize the improvement of resistance to BB and BLS. However, *Xoc* is known to suppress *R* gene-mediated resistance [15,16,33]. Our previous studies revealed that the *E* gene *Xa23* has the broadest and highest resistance to BB [12]. To investigate whether the *Xa23*-mediated resistance is efficient for controlling BLS, here, we generated a new version of *Xa23* (designated as *Xa23p1.0*) by inserting a 17 bp EBE into the *Xa23* promoter (Figure 1), which can specifically trap the conserved TALEs from multiple *Xoc* strains [34,35]. This work provides an alternative strategy to exploit the full potential of the *E* gene-mediated disease resistance against multiple diseases in rice.

## 2. Results

### 2.1. Generation of EBE-Modified Xa23 Transgenic Rice Plants

**Figure 2:**
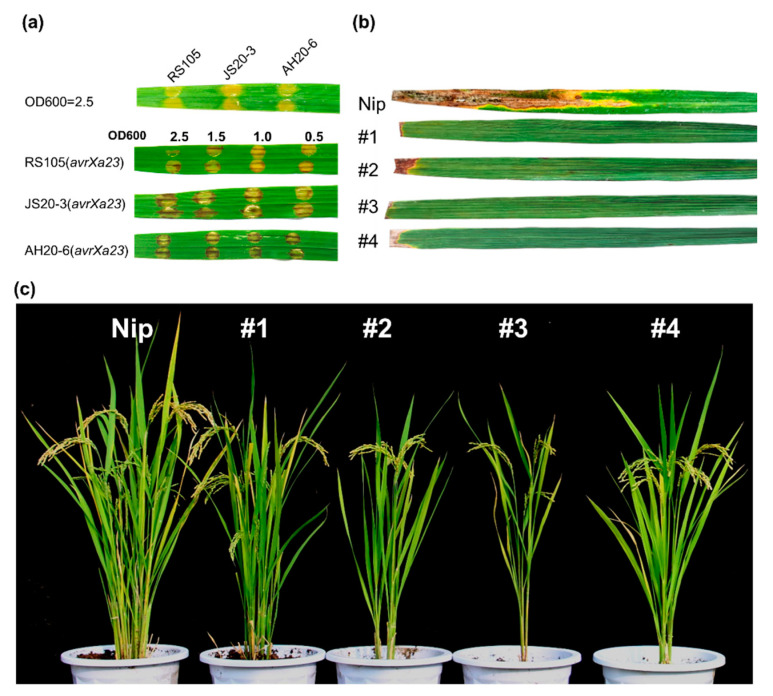
Generation of *Xa23p1.0* transgenic rice. (**a**) The phenotype of CBB23 was photographed 5 days after infiltration with transformed *Xoc* strains containing empty vector and pHZWavrXa23 at different OD600 values. (**b**) Disease phenotypes of *Xa23p1.0* transgenic T_2_ plants and wild-type Nipponbare (Nip) at tillering stage inoculated with *Xoo* strain PXO99^A^ by leaf-clipping method. (**c**) Phenotypes of the T_2_ plants at mature stage in Beijing field trial.

Our previous studies revealed that the *E* gene *Xa23* performed the broadest resistance against BB, because the cognate TALE (AvrXa23) conservatively exists in virtually all *Xoo* strains tested [12]. However, some avirulent TALEs, e.g., AvrXa7 and AvrXa10, failed to induce the resistance in rice cultivars with the corresponding *R* genes after being introduced into *Xoc* strains [33]. To preliminarily test if the avirulent TALE can induce strong defense responses in *Xa23*-containing rice varieties, we transformed pHZWavrXa23 into three *Xoc* strains (Table S1), and the resulting engineered *Xoc* strains were, subsequently, inoculated into CBB23 seedlings via the needleless syringe method. For the quantitative suppression characteristics of *Xoc*, transformants were inoculated at different cell concentrations [16,33]. The control groups, *Xoc* strains carrying the empty vector, caused water-soaking symptoms in CBB23 at the maximum concentration (OD600 = 2.5) (Figure 2a). Additionally, the transformants carrying pHZWavrXa23 at different concentrations (OD600 = 0.5–2.5) all successfully developed brown spots, which is the typical symptom of HR, as a result of incompatible interactions, and bacterial spread was restricted in inoculated areas three days after infiltration (Figure 2a), implying that *Xa23* can be developed into an artificial *E* gene against BLS. Based on the mechanism of the *Xa23*-mediated disease resistance, we speculated that the addition of EBE_Tal2g_, a 17 bp nucleotide sequence recognized by the conserved TALE member Tal2g_BLS256_ from *Xoc* [36,37], into the promoter of *Xa23* would achieve broad-spectrum resistance for both BB and BLS. To this aim, we amplified the *Xa23* locus from CBB23 and generated a new *E* gene, designated as *Xa23p1.0*, by inserting the EBE_Tal2g_ upstream of the AvrXa23 binding site in the *Xa23* promoter (Figure 1).

**Figure 3:**
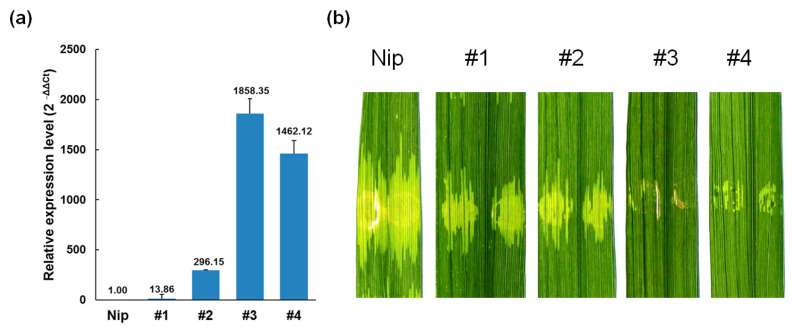
The basal levels of expression of *Xa23p1.0* in transgenic rice plants. (**a**) The background expression of *Xa23* in the T_2_ plants of *Xa23p1.0* transgenic rice. Relative expression levels were shown as a bar graph. (**b**) Disease reactions of *Xa23p1.0* transgenic rice leaves to *Xoc* strain RS105, which was conducted by needleless syringe infiltration at OD600 = 1.0.

The engineered *Xa23p1.0* was, subsequently, cloned into the transformation-competent artificial chromosome vector pYLTAC380H [38] and introduced into rice variety Nipponbare (Nip) through an Agrobacterium-mediated transformation. Four (#1, #2, #3, and #4) independent *Xa23p1.0*-postitive transgenic T_0_ plants were regenerated from ~1200 calli and found to be resistant to the *Xoo* representative strain, PXO99^A^ (Table S1). In subsequent T_1_ and T_2_ generations, the transgenic-positive plants derived from the T_0_ plants retained BB resistance (Figure 2b), but showed a somewhat stunted growth, characterized by a shorter plant height and less tillers (Figure 2c). We examined the T_2_ plants using q-PCR and found that the *Xa23p1.0* transgenic plants had differential levels of constitutive *Xa23* expression (Figure 3a). This was consistent with the speculation that the leaky expression of an *E* gene could cause stress-related phenotypes in transgenic plants [9,32].

### 2.2. Xa23p1.0 Transgenic Rice Plants Exhibit Resistance to Xoc and Magnaporthe oryzae

When the corresponding avirulence (*avr*) gene, *avrXa23*, was expressed from a high-copy plasmid, we observed a strong HR in CBB23 (Figure 2a). However, we still needed to firstly investigate whether the *Xa23*-mediated resistance induced by the endogenous *avr* gene on the chromosome was efficient for BLS. For that, the resistance suppression using *Xoc* was quantitative [16,33]. Here, RS105, the representative *Xoc* strain, was firstly used to verify the resistance of *Xa23p1.0* in the positive T_2_ plants derived from the four transgenic rice lines, via needleless syringe infiltration (Figure 3b). The BLS lesions in the T_2_ plants of *Xa23p1.0*-transgenic rice were obviously shorter than that in the control Nipponbare. However, the inoculated spots did not turn brown in the transgenic rice plants, which was different from spots caused by RS105/pHZWavrXa23 in CBB23. The results revealed that suppression of *Xa23*-mediated resistance existed in rice-*Xoc* interactions. In addition, the water-soaking symptoms caused by RS105 in the transgenic lines #1 and #2 seemed stronger than that in line #3 and #4 (Figure 3b), demonstrating a high background expression that seemed to confer a strong resistance in rice against *Xoc*. It also hinted that *Xoc* produced a dose-dependent suppression of the *Xa23*-mediated disease resistance response.

**Figure 4:**
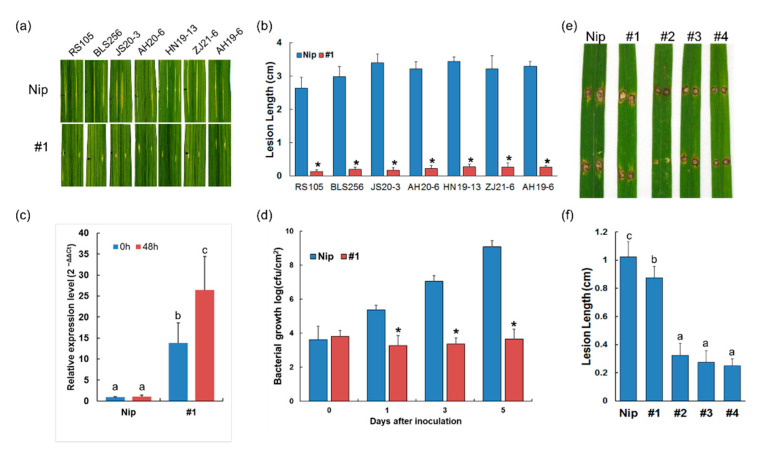
Ectopic expression of *Xa23* enhances resistance to multiple rice diseases. (**a**) BLS disease phenotypes of the T_2_ plants at tillering stage inoculated with *Xoc* strains with leaf pin-pricking method at OD600 = 1.0. (**b**) BLS lesion lengths in the T_2_ plants. Column height shows mean lesion length (cm), and error bars indicate the standard deviation of three replicates. (**c**) The expression of *Xa23* was evaluated by qRT-PCR at 48 h post inoculation with *Xoc* strain RS105. Data are means ± SD of four biological replicates. (**d**) Bacterial populations in the leaves challenged with *Xoc* strain RS105. *p* value were calculated by *t*-test, * *p* < 0.05. (**e**) Blast disease phenotypes in the T_2_ plants at seeding stage and 7 days after punch inoculation with *M*. *oryzae* strain Rb-17 (~5 × 10^5^ spore/mL). (**f**) Blast lesion lengths caused by *M*. *oryzae* strain Rb-17. For (**c**,**f**), different letters indicate a statistically significant difference between values (*p* < 0.05).

To accurately evaluate *Xa23p1.0* resistance to BLS and test the effectiveness of combining EBE_Tal2g_ and *Xa23*, five highly virulent *Xoc* strains (Table S1), together with RS105 and BLS256, were used to inoculate the T_2_ plants derived from the T_0_ plant #1, whose basal level of expression of *xa23*/*Xa23* was similar to that in Nipponbare. Disease assay results showed that the T_2_ plants were highly resistant to all the *Xoc* strains, characterized by significantly shorten lesions (Figure 4a,b). Consistent with the resistance phenotype, the *Xa23* expression was significantly induced in the T_2_ plants when challenged with *Xoc* (Figure 4c). The population growth of RS105 was significantly reduced in the #1 plant relative to the control Nipponbare (Figure 4d). These results indicated that EBE_Tal2g_ in the *Xa23p1.0* promoter successfully trapped the conserved TALEs from *Xoc*. In short, the above data clearly demonstrated that *Xa23p1.0* conferred broad resistance to both BB and BLS in rice.

The defense pathway against fungal pathogens may overlap with the defense pathway against bacterial pathogens in rice. Additionally, no studies have confirmed that the *E*-gene-mediated disease resistance is effective against fungal pathogens. Blast caused by the fungus *Magnaporthe oryzae* is another destructive disease of rice. To inspect whether *E* gene expression is valuable for resistance against fungal pathogens, we conducted blast resistance assays on the *Xa23p1.0* transgenic plants. Therefore, Rb-17, a virulent *M*. *oryzae* isolate (Table S1), was punch-inoculated on detached leaves from the abovementioned T_2_ plants at the seedling stage. The blast lesions caused by Rb-17 were obviously shorter in the transgenic plants compared with the wild type (Figure 4e,f). It is noteworthy that the transgenic lines derived from T_0_ plants #2, #3, and #4 with a higher leaky expression of *Xa23* conferred a stronger resistance to *M*. *oryzae* (Figure 4e,f). These results suggested that *Xa23*-mediated disease resistance could also be effective against rice blast. Meanwhile, we found that the gene expression level correlated with the intensity of the BLS and blast resistance.

### 2.3. The Stressed Phenotypes of Xa23p1.0 Transgenic Plants Were Relieved in the Sanya Field Trial

**Figure 5:**
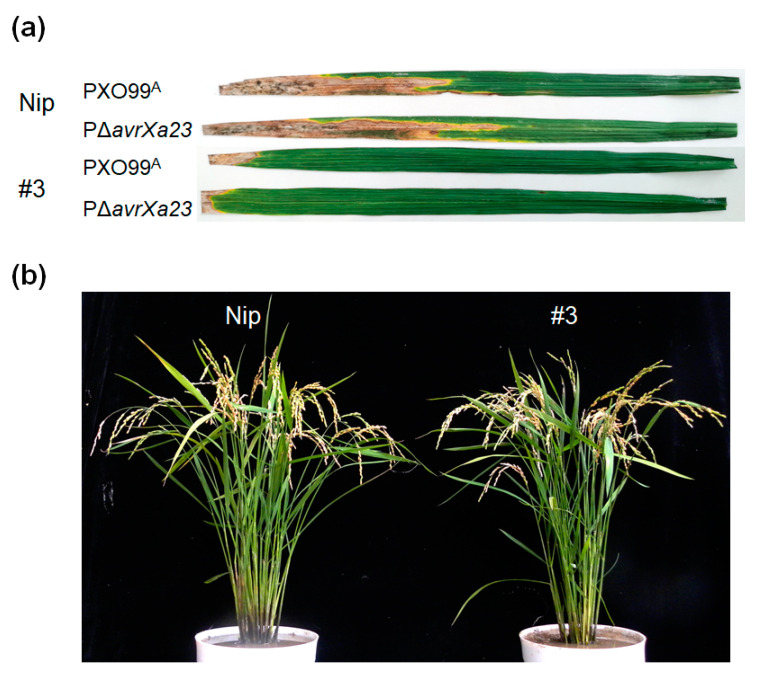
Leaky expression of *Xa23* led to over-response of the immune system. (**a**) Disease phenotypes of T_2_ plants of line #3 and wild-type Nipponbare (Nip) at tillering stage inoculated with PXO99^A^ and *avrXa23* deletion mutant PΔavrXa23 by leaf-clipping method at OD600 = 1.0. (**b**) Phenotypes of the T_2_ plants of line #3 at mature stage in Sanya field trial.

As mentioned previously, the *Xa23p1.0* transgenic rice plants exhibited stress phenotypes and yield loss due to the leaky expression of *Xa23*. The leaky expression of the *E* gene led to an over-response of the immune system in plants. To investigate the resistance intensity of stressed plants, we performed pathogenicity assays using an *avrXa23*-deleted mutant, PΔavrXa23, through leaf-clip inoculation in T_2_ plants derived from the transgenic line #3, which had the highest leaky expression level of *Xa23p1.0* based on the q-PCR results. PΔavrXa23 is an *avrXa23* deletion mutant derived from PXO99^A^, and has high virulence in *Xa23*-containing rice. PΔavrXa23 and the wild type caused similar lesion lengths in the susceptible control Nipponbare, while the T_2_ plants of #3 exhibited strong resistance to both the virulent and avirulent *Xoo* strains (Figure 5a). These results indicated that the activated immunity in the #3 plants could guard hosts from multiple pathogen infections.

Although the *Xa23p1.0* transgenic rice successfully acquired a stronger disease resistance, over-reactive immune responses could induce a negative impact on agronomic characters. To evaluate the influence for the related agronomic characters, we conducted two independent field trials of T_2_ plants from #3 and the wild-type Nipponbare in Beijing (BJ) and Sanya (SY) in 2021. Most obviously, the plant height, number of tillers, number of panicles, and seed weight per plant were drastically decreased for excessive immune response in the two field trials. The main environmental difference between our two experimental stations was the monthly average temperature. The temperature of Sanya station was slightly higher than that in Beijing, and the total growth period of the rice plant is shorter in high temperatures. Additionally, the stressed phenotypes were more remarkable in the Beijing field trial in the summer (June through October). The T_2_ plants from #3 only had 33.3% tillers and 31% panicles in comparison to the wild-type Nipponbare (Figure 2c and Table 1). A reduction in tiller numbers is the major cause for serious yield losses (about 53.1%) per plant, and the plant height and seed setting rate also significantly decreased. However, in the field trial in a warm climate (Figure 5b), the phenotypic differences between the #3 T_2_ plants and Nipponbare were narrowed down. The number of panicles and seed weight per plant of the transgenic rice were recovered to 73.8% and 77.7% of the wild type, respectively. Particularly, the plant height of the transgenic rice was close to that of the wild type. These findings indicated that the stressed phenotypes of the *Xa23p1.0* transgenic line, #3, were significantly relieved in the field trial in the tropical region. In addition, although the transgenic rice exhibited intense stressed phenotypes, the thousand seed weight was not obviously different between the #3 T_2_ plant and Nipponbare. We also found that the seed setting rate of the #3 T_2_ plant and Nipponbare decreased sharply in the Sanya field trial.

## 3. Discussion

Unlike the nucleotide-binding leucine-rich repeat (NLR)-type *R* genes, which are common and abundant [39], a few *E* genes (only six cloned to date) consist of a unique type of plant *R* genes. These *E* genes are TALE-dependent, and they can specifically trap the TALEs of pathogens and trigger an intense defense reaction characterized by HR in the host. This strong resistance is a result of programed cell death induced by the *E* gene expression that is only activated upon the binding of a TALE to the EBE located in the *E* gene promoter during a pathogen infection [2]. According to the TALE–DNA binding principle, artificial EBE sequences can be integrated into promoters of *E* genes to generate broad-spectrum resistance by trapping expected TALEs. However, the *E* genes are often expressed constitutively using the conventional transgenic approach.

CRISPR-Cas9-mediated precise homology-directed repair (HdR) provides an ideal strategy to modify the promoters of *E* genes and their alleles at a native locus for preventing leaky expression [40]. The other notable thing is that endogenous *E* gene (or the allele) loci are absent in some rice varieties; for example, Nipponbare does not contain *Xa27* and its allele [41]. The exploration of more valuable members is important for *E* gene utilization in biological breeding for resistance to BB and BLS. It has been reported that the insertion of EBEs into the *Xa27* promoter conferred sufficiently strong resistance to *Xoo* and *Xoc* in rice [30]. However, the suppression of host resistance was observed in multiple plant–pathogen interaction systems, such as the rice–*Xoc* interaction system. To evaluate the prospects of applying *Xa23* in controlling BLS, we developed an EBE-amended *Xa23*, designated as *Xa23p1.0*. Afterwards, *Xa23p1.0* was integrated with EBE sequences targeting conserved TALEs in both *Xoo* and *Xoc*, and conferred broad-spectrum resistance to two bacterial diseases in rice. The results also indicated that *Xoc* could not successfully suppress *Xa23*-meidated resistance in rice.

HR-like cell death is the most effective plant immune response restricting pathogen invasion and growth [42,43]. The *E*-gene-mediated HR has often been accompanied by typical brown spots at the inoculation sites in rice. When the wild *Xoc* isolate was injected into the *Xa23p1.0* transgenic rice plants, the plant leaves exhibited resistance against pathogens, but the inoculated spots did not appear to like what was induced by AvrXa23 expressed from a high-copy plasmid in *Xoc* (Figure 2a). Simultaneously, we found that the BLS symptoms were less severe in the *Xa23p1.0* higher leaky expression plant (Figure 3b and Figure 4a). These observations reinforced the view that rice resistance to BLS is quantitative, and the suppression of host innate immunity generally exists in *Xoc*–rice interactions. The results hinted that a high-level expression of *Xa23p1.0* could defeat the immunosuppressive effects of pathogens. To acquire strong resistance for BLS, multiple tandem EBEs can be integrated into promoters to trap more TALE members to induce a high-level expression of the *E* gene.

Meanwhile, the *Xa23p1.0* transgenic rice provided opportunities to test the *Xa23*-meidated resistance to other pathogens in rice. Virulence assays on rice seedlings indicated that the leaky expression of *Xa23p1.0* enhanced host resistance to *M*. *oryzae*. The over-activation of immune response conferred a strong host resistance to multiple pathogens without a specific recognition mechanism in the *E*-gene-mediated resistance. However, the leaky expression of *Xa23p1.0* stressed the phenotypes in transgenic rice, leading to an over-response of the immune system. Notably, based on the agronomic data in Beijing and Sanya, we found that the stressed phenotypes of *Xa23p1.0* transgenic rice were relieved in the field trial in the tropical region. It was reported that *Xa7* conferred a stronger rice resistance at higher temperatures. There were differences in multiple climatic factors between Beijing and Sanya. Does that imply that plants have some regulatory mechanisms to adjust the negative influence of an immune response under high temperatures? In the future, we would like to study the effect of temperature in the *E*-gene-mediated resistance.

*E* genes can achieve broad resistance through integrating pathogen-responsive *cis*-elements, such as EBEs, into the promoters of *E* genes or their alleles to sense multiple pathogen effectors. The *E*-gene-mediated immune response can protect the host against bacterial and fungal pathogens. CRISPR-Cas9-mediated precise homology-directed repair might be an ideal strategy to create resistant germplasms, because some unknown elements should exist at a native locus to control the background expression of *E* genes. The low basic levels of expression of *E* genes avoid the damage caused by the over-response of the immune system. In conclusion, our work successfully expanded the *Xa23* resistance spectrum from BB to BLS by adding EBE_Tal2g_ in the promoter region. Furthermore, we demonstrated that the *Xa23*-meidtaed defensive reaction was also effective against fungal *M*. *oryzae*, the causal agent of rice blast. These findings imply that the identification of pathogen-responsive *cis* elements with a low background expression and efficient pathogen induction activity might be a key factor for exploiting the full potential of *E* gene resistance in the future.

## 4. Materials and Methods

### 4.1. Plant Materials and Bacterial Strains

For seedling inoculation and field trail, seeds of Nipponbare and *Xa23p1.0* transgenic plants were germinated in Petri dish for 5 days in a 30 °C dark incubator. Seedings were transplanted to a plastic box (61 × 42 × 15 cm) with Pindstrup substrate (P8988, Ryomgaard, Denmark). Plants were grown in a greenhouse at 26 to 32 °C with a light and dark cycle of 14 and 10h, until they were at five-leaf stage. *Escherichia coli* strains were grown in Luria–Bertani (LB) medium supplemented with appropriate antibiotics at 37 °C. *Agrobacterium tumefaciens* strains were grown at 30 °C under the dark. All *Xoo* and *Xoc* strains were grown at 28 °C in nutrient broth (NB) (1% peptone, 0.5% yeast extract, 1% sucrose, and 1.5% agar). Antibiotics (Sangon, Shanghai, China) were used at the following concentrations if required: 100 μg/mL ampicillin, 25 μg/mL rifampicin, 25 μg/mL kanamycin, and 100 μg/mL spectinomycin.

### 4.2. Field Experiment

To evaluate the agronomic characteristics under normal field conditions, both Nipponbare and *Xa23p1.0* transgenic rice were grown to five-leaf stage in greenhouse (see Section 4.1). Then, the seedings were planted in a three-row plot with ten plants per row, applying 20 × 16 cm spacing at Hainan experimental station (18.396448 N, 109.203067 E) of Institute of Crop Sciences from January to May and Shunyi experimental station (40.237433 N, 116.570354 E) of Institute of Crop Sciences from June to October and January to May in 2021. Ten plants in the middle row of each line were sampled for the agronomic characteristics investigation.

### 4.3. Plant Inoculations and Disease Assays

For isolating new *Xoc* strains, rice leaves with typical BLS symptoms were firstly collected from paddy in southern China from 2018 to 2021. After surface sterilization, the leaves were crushed in microcentrifuge tubes containing 500 μL distilled H_2_O using a plastic pestle. Then, the bacterial suspension was serially diluted and plated onto NA plates incubated at 28 °C for 4 days. The suspected *Xoc* clones were selected, purified, and identified using specific primers with colony PCR amplification (Table S2) [14].

For *Xoo* and *Xoc* inoculation, bacterial cells were collected from culture at low-speed (4000 r.p.m.) centrifugation, washed twice and suspended in sterile water. The suspensions were adjusted to required optical density, e.g., OD600 = 1.0 (approx. 1 × 10^9^ cell/mL), as described previously [21]. The adult rice plants were inoculated in *Xoo* suspension by the leaf-clipping method in the fields of our experimental station and *Xoc* suspension by needleless syringe infiltration method in greenhouse and pin-pricking method in fields of our experimental station. Lesions were photographed and measured 15 days after inoculation (DAI) for leaf-clipping and pin-pricking inoculation. For syringe infiltration inoculation, lesions were photographed and measured 5 DAI. 

To measure bacterial populations, infiltrated areas of rice leaves were sterilized and crushed as above. Then, samples were diluted serially in sterile water and spread onto NA plates. Plates were inoculated at 28 °C until single colonies could be counted. Results were displayed as the mean and standard deviation of all measurements for three replicates. For *M. oryzae* inoculation, the punch inoculation method was conducted as described previously [44]. Briefly, isolate Rb-17 was cultured on oat meal agar medium for 14 days for generating spores. Spores were collected by washing the agar cultures with sterile water (containing 1% Tween 20), and the spore concentration was adjusted to ~5 × 10^5^ spore/mL. Detached seedling leaves were wounded with a hole-punch. An amount of 7 μL spore suspension was applied to the injured area, and then the inoculated leaves were placed in sterile water that contained 0.1% 6-Benzylaminopurine to keep them moist. The inoculated leaves were kept in darkness at 28 °C for 24 h before they were transferred to a controlled growth chamber. Lesions were photographed and measured when significant difference was observed.

The disease assays were performed at least twice. Three replicates with approximately 3 leaves from three to five plants per replicate were inoculated per strain. One-way analysis of variance (ANOVA) statistical analysis was conducted on all measurements. Tukey’s honestly significant difference test was used for post-ANOVA pair-wise tests for significance, set at 5% (*p* < 0.05).

### 4.4. Genes and Constructs

The upstream (~1299 bp) and downstream (~940 bp) fragments of *Xa23* were amplified from CBB23 gDNA, and the 17 bp nucleotide sequence of EBE_Tal2g_ was inserted into the native promoter of *Xa23* through Gibson assembly method (C113-01, Vazyme, Nanjing, China) to generate *Xa23p1.0* (Table S2). Then, *Xa23p1.0* was ligated into pYLTAC380H and introduced into Nipponbare using *Agrobacterium*-mediated transformation described previously [12].

### 4.5. qRT-PCR Analyses

To analyze the gene induction, three-week-old rice seedlings were selected for *Xo* (OD600 = 1.0) inoculation with the needleless syringe method. The treated and untreated leaves were harvested and grinded in Trizol (Thermofisher, Waltham, USA) to extract total RNAs. Then, the treated RNA samples were reversely transcribed into cDNA by reverse transcription Kit (TIANGEN, Beijing, China). qRT-PCR was performance on ABI 7500/7500 Fast Real-Time PCR system using SYBR^®^ Premix ExTaqTM II kit (TaKaRa, Dalian, China). The reaction volume was 20 μL, containing 10 μL 2× SYBR Green Master Mix, 2 μL cDNA, 0.4 μL 50× ROX Reference Dye II, 0.8 μL forward and reverse primers (10 μmol L^−1^), and 6 μL RNase-free H_2_O. The PCR procedure was predenaturation at 95 °C for 2 min, 95 °C for 15 s, 60 °C for 30 s, and 40 cycles. The rice Actin gene was set as internal reference gene (Table S2). The 2^−∆∆Ct^ method was used as a relative quantification strategy for data analysis, and three biological replicates were measured for each sample.

## Figures and Tables

**Table 1:** Agronomic characteristics of Nipponbare and *Xa23p1.0* transgenic rice in Beijing and Sanya field trials.

	Rice Lines	Plant Height (cm)	Number of Tillers	Number of Panicles	Panicle Length (cm)	Seed Setting Rate (%)	Thousand Seed Weight (g)	Seed Weight per Plant (g)
BJ	Nip	67.88 ± 4.91 ^c^	22.50 ± 1.66 ^c^	21.00 ± 1.80 ^b^	17.34 ± 0.95 ^c^	79.45 ± 2.36 ^c^	25.33 ± 0.87 ^a^	26.21 ± 0.95 ^c^
	#3	52.75 ± 2.45 ^a^	7.50 ± 0.87 ^a^	6.50 ± 1.22 ^a^	12.93 ± 1.17 ^a^	58.36 ± 4.27 ^a^	26.25 ± 0.48 ^a^	12.28 ± 2.19 ^a^
SY	Nip	64.80 ± 1.55 ^c^	26.30 ± 1.73 ^d^	25.20 ± 2.32 ^c^	16.40 ± 0.81 ^bc^	69.69 ± 3.99 ^b^	27.37 ± 0.92 ^a^	25.80 ± 3.13 ^c^
	#3	59.00 ± 2.45 ^b^	19.50 ± 2.06 ^b^	18.60 ± 2.41 ^b^	16.05 ± 0.56 ^b^	68.87 ± 6.93 ^b^	26.57 ± 1.01 ^a^	20.05 ± 1.54 ^b^

Results are shown by average data (the mean ± SD) from ten plants of each line. Different letters indicate a statistically significant difference between values (*p* < 0.05). Beijing, BJ; Sanya, SY.

## Data Availability

The data presented in this study are available on request from the corresponding author.

## Supplementary Materials

The following supporting information can be downloaded at: https://www.mdpi.com/article/10.3390/ijms23126545/s1 [45].
