# Supplementary material for: Ectopic Expression of Executor Gene Xa23 Enhances Resistance to Both Bacterial and Fungal Diseases in Rice
Source: Int J Mol Sci. 2022 Jun 11;23(12):6545. doi: 10.3390/ijms23126545 (PMC9224217; doi:10.3390/ijms23126545)
Supplement: Supplementary file 1 [file ijms-23-06545-s001.zip › ijms-1719862-supplementary.pdf]

Supplementary Table S1. List of plasmids and pathogen strains used in this study

Strains or Plasmids	Relevant characteristics	Reference/Source
**Strains**		
Xanthomonas oryzae pv. *oryzae*		
PXO99^A	Philippine race 6, GenBank: CP000967.2	[1]
PΔavrXa23	*avrXa23* mutant of PXO99^A, Tal9 cluster was deleted	This study
Xanthomonas oryzae pv. *oryzicola*		
RS105	Chinese strain, GenBank: CP011961.1	[2]
BLS256	Philippine strain, GenBank: CP003057.2	[2]
JS20-3	Strain isolated from Jiangsu in 2020	This study
AH20-6	Strain isolated from Anhui in 2020	This study
HN19-13	Strain isolated from Hainan in 2019	This study
ZJ21-6	Strain isolated from Zhejiang in 2021	This study
AH19-6	Strain isolated from Anhui in 2019	This study
Magnaporthe grisea		
Rb-17	Strain isolated from Northeast of China	This study
Agrobacterium tumefaciens		
EHA105	C38(Rif^R) Ti pEHA105(pTiBo542DT-DNA) Succinamopine, for rice transfornation	This study
**Plasmids**		
pHZWavrXa23	pHM1 expressing *avrXa23* from PXO99^A in pZW	[3]

Supplementary Table S2. Primers and sequence information.

Primer	Sequence (5′ to 3′)	Usage
Xa23F	5′-GTAGAACAGCATGACCGAGAGAC-3′	*Xa23* (KP123634.1) gene-specific primers for q-PCR
Xa23R	5′-GTAGCCGGTATACACATGATCCTC-3′	
UbiF	5′ -GCTCCGTGGCGGTATCAT-3′	q-PCR primer for ubiquitin gene as internal controls
UbiR	5′ -CGGCAGTTGACAGCCCTAG-3′	

References

1. Salzberg, S.L., Sommer, D.D., Schatz, M.C., Phillippy, A.M., Rabinowicz, P.D., Tsuge, S., Furutani, A., Ochiai, H., Delcher, A.L., Kelley, D., et al. Genome sequence and rapid evolution of the rice pathogen *Xanthomonas oryzae* pv. *oryzae* PXO99[A]. *BMC Genomics.* **2008**, *9*, 204. doi: 10.1186/1471-2164-9-204.
2. Wilkins, K.E.; Booher, N.J.; Wang, L.; Bogdanove, A.J. TAL effectors and activation of predicted host targets distinguish Asian from African strains of the rice pathogen *Xanthomonas oryzae* pv. *oryzicola* while strict conservation suggests universal importance of five TAL effectors. *Front. Plant Sci.* **2015**, *6*, 536.
3. Wang, C.L.; Zhang, X.P.; Fan, Y.L.; Gao, Y.; Zhu, Q.L.; Zheng, C.K.; Qin, T.F.; Li, Y.Q.; Che, J.Y.; Zhang, M.W.; et al. XA23 Is an Executor R Protein and Confers Broad-Spectrum Disease Resistance in Rice. *Mol. Plant* **2015**, *8*, 290–302.
